# Theory and Practice of the Movement-Cure

**Published:** 1861-03

**Authors:** 


Theory and Practice of the *Movement-Cure:* or the Treatment of Lateral Curvature of the Spine; Prolapsus; Indigestion; Constipation; Consumption; Angular Curvatures, and other Deformities; Diseases Incident to Women; Derangements of the Nervons System; and other Chronic Affections; by the Swedish System of *Localized Movements.* By Charles Fayette Taylor, M. D.; with illustrations. Philadelphia: Lindsay & Blakiston, 1861.

This is a small octavo volume, containing 295 pages, done up in excellent style. It contains the most full and interesting exposition of systematic muscular exercise, and its effects in preserving health and curing diseases, with which we are acquainted. The subject is one of much importance, and hitherto, altogether too much neglected. The present work fills a vacancy in our Medical Literature, and may be read with much profit by every practicing physician. For sale by S. C. Griggs & Co., Chicago.

